# Supplementary material for: Age-specific genomic and transcriptomic variation reveals limited evidence for cis-regulatory interactions modulating aging in Saccharomyces cerevisiae
Source: bioRxiv. 2025 Dec 14:2025.12.12.689579. Preprint. [Version 1] doi: 10.64898/2025.12.12.689579 (PMC12713674; doi:10.64898/2025.12.12.689579)
Supplement: Supplement 6 [file media-6.pdf]

Gene List	*Category*	*Term*	*P-value*	*Associated features*
**Genome**	Component	cell periphery	5.49E-07	AIM44, HKR1, YHL026C, SIM1, FKS1, YPS6, CRH1, JEN1, HSC82, TOR2, HXT3, WSC3, FRE3, HIP1, THI7, BBC1, DAN4, TDH3, STE6, GTS1, AGA1, PHO90, TIR1, OPT2, YNL190W, SAC1, FIG2, TIR2, MTL1, SEC3, PGA3, ADH1, YBR067C, SUC2, NUM1, FLO11
		cell wall	1.73E-03	YNL190W, DAN4, FIG2, TDH3, AGA1, TIR1, YPS6, CRH1, SIM1, YBR067C
		fungal-type cell wall	1.73E-03	AGA1, TIR1, CRH1, YPS6, SIM1, YBR067C, YNL190W, DAN4, FIG2, TDH3
		external encapsulating structure	1.73E-03	SIM1, CRH1, YPS6, YBR067C, AGA1, TIR1, FIG2, TDH3, YNL190W, DAN4
		plasma membrane	1.10E-02	MTL1, PGA3, THI7, ADH1, FRE3, HIP1, STE6, FLO11, TDH3, HKR1, AIM44, PHO90, JEN1, FKS1, OPT2, WSC3, HXT3, HSC82, TOR2
		golgi apparatus	4.00E-02	YKT6, MNN2, MNN5, MNN1, MNN4, STE6, SAC1, MNN11, YND1, GEA2, OPT2, SPF1
		golgi cisterna	8.79E-02	MNN11, SAC1, GEA2, OPT2
	Process	protein glycosylation	1.10E-02	MNN2, MNN4, YND1, MNN1, MNN11, MNN5, ALG7, PMI40
		macromolecule glycosylation	1.10E-02	ALG7, PMI40, MNN11, MNN1, MNN5, YND1, MNN2, MNN4
		glycosylation	1.30E-02	ALG7, PMI40, MNN1, MNN11, MNN5, YND1, MNN4, MNN2
		glycoprotein biosynthetic process	1.80E-02	MNN5, MNN1, MNN11, PMI40, ALG7, MNN2, MNN4, YND1
		glycoprotein metabolic process	4.30E-02	YND1, MNN4, MNN2, PMI40, ALG7, MNN5, MNN1, MNN11
	Function	NA	NA	NA
**Transcriptome**	NA	No Significant GO-terms	NA	NA
**Shared Genes**	Process	Iron Coordination entity transport	3.13E-02	DNM1, FIT2
		iron ion transport	9.98E-02	FIT2, DNM1
	Component	NA	NA	NA
	Function	NA	NA	NA

Supplementary Table 3: GO-terms returned for significant results. These GO-terms were overrepresented among our candidate variant lists for the genome and within genes that were present in both genomic and transcriptomic variant lists ("Shared Genes", Table 1). GO-term analysis was conducted using the Saccharomyces Genome Database Gene-Ontology term finder with an FDR correction and $p<0.05$. No GO-terms were returned for the list of significantly differentially expressed transcripts ($p<0.1$, FDR correction).
