# Supplementary material for: Age-specific genomic and transcriptomic variation reveals limited evidence for cis-regulatory interactions modulating aging in Saccharomyces cerevisiae
Source: bioRxiv. 2025 Dec 14:2025.12.12.689579. Preprint. [Version 1] doi: 10.64898/2025.12.12.689579 (PMC12713674; doi:10.64898/2025.12.12.689579)
Supplement: Supplement 7 [file media-7.pdf]

Peak	*Chromosome*	*Elevated haplotype*	*Reduced haplotype*	*Associated features*
A	5	DBVPG6765	DBVPG6044	MNN1, MIT1,YND1, PMP2, WBP1, NOP16, IRC22, PMI40, TMA20, NUG1, TMA20, SEC3, SNR14, TIR1, YER010C
B	10	YPS128	DBVPG6044	MNN5, SWI3, SWE1, RFA3, RPS22A, ATG36, YJL181W, YJL182C, ELO1, ATG27, ATP12, YJL163C, YJL202C, ACO2, LAA1, RPS14B, YJL171C, SOP4, YJL182C, PHO90, UBP12, MNN11, YJL197C-A, RPS22A, CPS1
C	11	Y12	DBVPG6765	PTK1, MNN4, TOR2, YKL202W, UBA1, TRP3, STE6, PEX1, SDS22, EAP1, EMC3, JEN1, CBT1, YKT6, URA1, DOA1, SAC1

Supplementary Table 4: Most significant peaks of haplotype differentiation. Elevated and reduced ancestral haplotypes for each peak are shown, along with genes that harbor genetic variants within peak regions.
