# Supplementary material for: Age-specific genomic and transcriptomic variation reveals limited evidence for cis-regulatory interactions modulating aging in Saccharomyces cerevisiae
Source: bioRxiv. 2025 Dec 14:2025.12.12.689579. Preprint. [Version 1] doi: 10.64898/2025.12.12.689579 (PMC12713674; doi:10.64898/2025.12.12.689579)
Supplement: Supplement 10 [file media-10.pdf]

Population	***Origin***	***n=***	***p-value***	***Mean bud scars in young fractions***	***Mean bud scars in aged fractions***
4S	Recombinant population	5	0.00389*	2.144	2.826
YPS128	North American oak forest	5	0.00091*	2.006	2.756
Y12	Japanese sake	5	0.0625	2.115	3.044
DBVPG6044	African palm wine	4	0.00453*	1.8	3.172
DBVPG6765	European wine	4	0.0284*	2.143	3.235

Supplementary Table 7: Bud scars in young and aged sorted fractions. The number of bud scars counted per cell in each population was averaged across biological replicates. P-values in the table come from within-population comparisons as evaluated by pairwise tests with t-tests (no shading) and Wilcoxon signed rank tests (shaded in gray), used as appropriate. Significant differences between young and aged cohorts within each population are indicated by an asterisk (*).
