# Supplementary material for: Age-specific genomic and transcriptomic variation reveals limited evidence for cis-regulatory interactions modulating aging in Saccharomyces cerevisiae
Source: bioRxiv. 2025 Dec 14:2025.12.12.689579. Preprint. [Version 1] doi: 10.64898/2025.12.12.689579 (PMC12713674; doi:10.64898/2025.12.12.689579)
Supplement: Supplement 11 [file media-11.pdf]

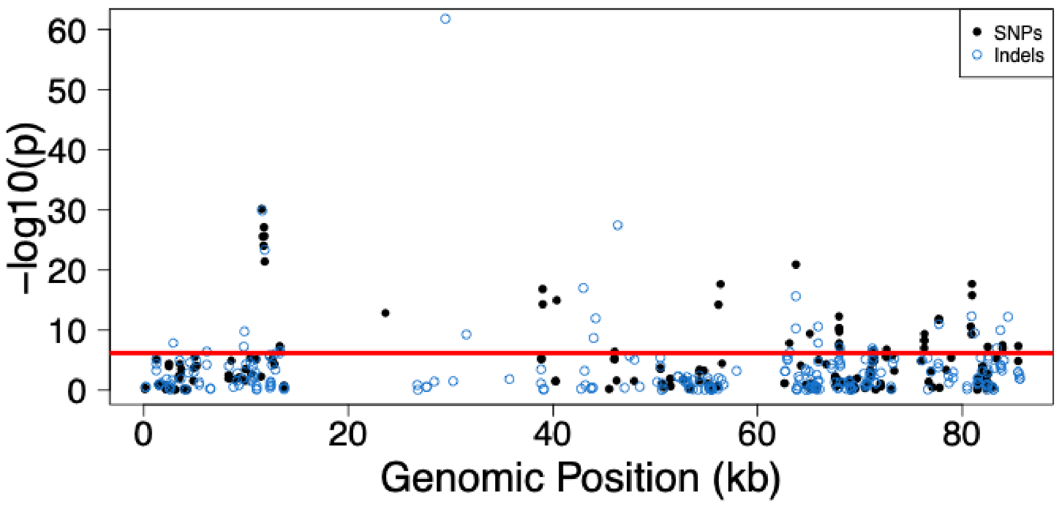

Supplementary Figure 1: Regions of genome differentiation within the mitochondria. SNPs are shown in black and indels are shown in blue. The red line shows a Bonferroni corrected α=0.05 threshold, generated using SNPs and indels from the whole genome.
