# Supplementary material for: Age-specific genomic and transcriptomic variation reveals limited evidence for cis-regulatory interactions modulating aging in Saccharomyces cerevisiae
Source: bioRxiv. 2025 Dec 14:2025.12.12.689579. Preprint. [Version 1] doi: 10.64898/2025.12.12.689579 (PMC12713674; doi:10.64898/2025.12.12.689579)
Supplement: Supplement 12 [file media-12.pdf]

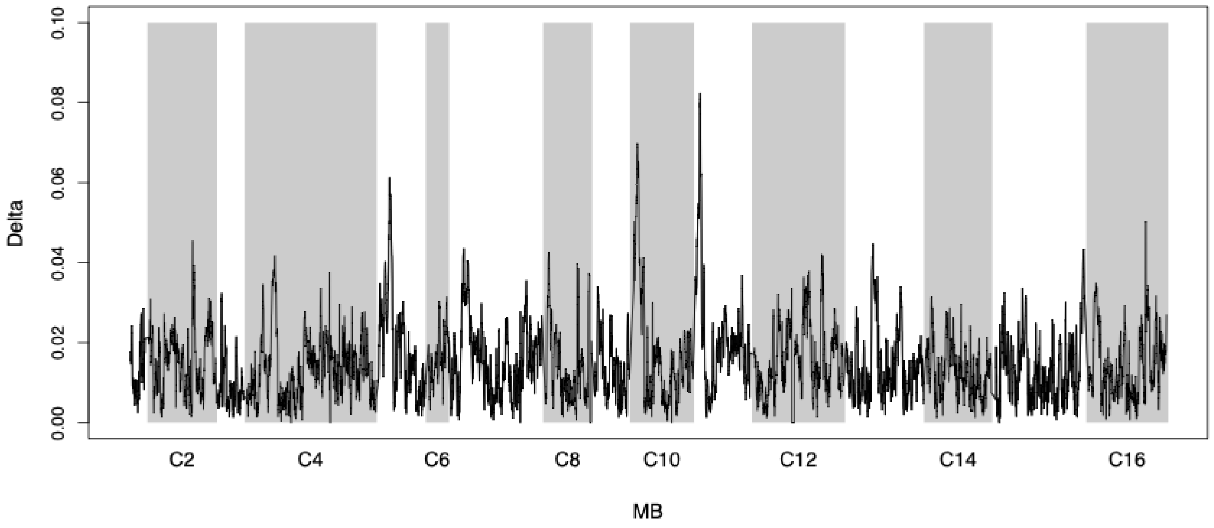


**Supplementary Figure 2:** Haplotype divergence ("D") across the genome. The average divergence between all ancestral haplotypes at each point in the nuclear genome is shown
