# Supplementary material for: Age-specific genomic and transcriptomic variation reveals limited evidence for cis-regulatory interactions modulating aging in Saccharomyces cerevisiae
Source: bioRxiv. 2025 Dec 14:2025.12.12.689579. Preprint. [Version 1] doi: 10.64898/2025.12.12.689579 (PMC12713674; doi:10.64898/2025.12.12.689579)
Supplement: Supplement 13 [file media-13.pdf]

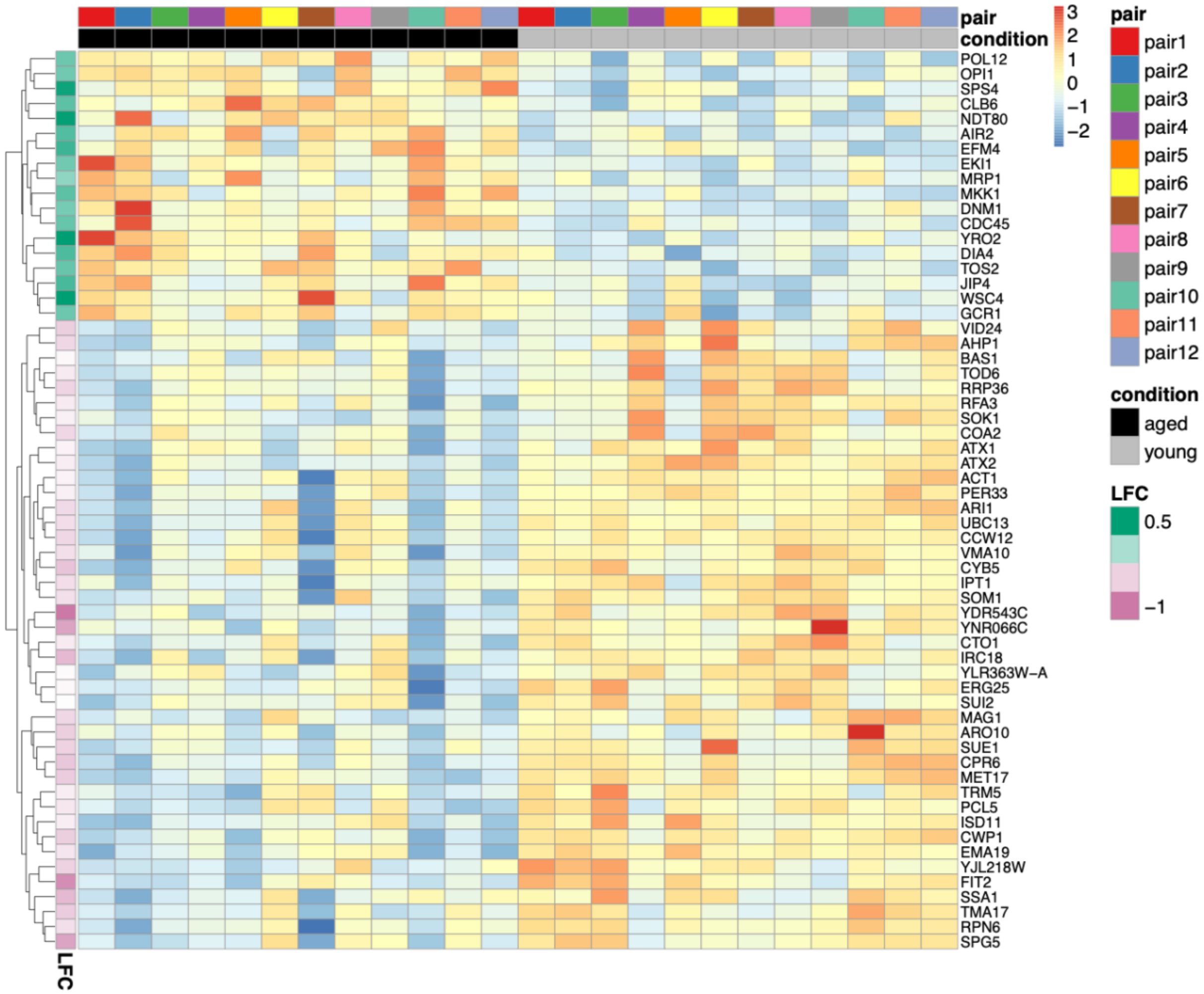

Supplementary Figure 3: Heatmap showing differential expression of significant genes ($p<0.1$). The top bar indicates the pairing structure of the data, and the second bar indicates the age of the replicate. The bar on the far left shows the average $\log_2$ fold change (LFC) of expression across replicates. Positive values of LFC (in green) indicate an increase in expression in the aged replicates.
